# The triponderal mass index as a measure of adiposity in pediatric survivors of acute lymphoblastic leukemia: a cross-sectional study

**DOI:** 10.1038/s41598-022-05236-5

**Published:** 2022-01-26

**Authors:** Alissa W. Zhang, John T. Wiernikowski, Carol Portwine, Lehana Thabane, M. Constantine Samaan

**Affiliations:** 1 Department of Pediatrics, McMaster University, 1280 Main Street West, HSC-3A57, Hamilton, ON L8S 4K1 Canada; 2 Division of Pediatric Endocrinology, McMaster Children’s Hospital, 1280 Main Street West, HSC-3A57, Hamilton, ON L8S 4K1 Canada; 3 Bachelor of Health Sciences (Honours) Program, Child Health Specialization, McMaster University, Hamilton, ON Canada; 4 Division of Pediatric Hematology/Oncology, McMaster Children’s Hospital, Hamilton, ON Canada; 5 Department of Health Research Methods, Evidence and Impact, McMaster University, Hamilton, ON Canada; 6 Department of Anesthesia, McMaster University, Hamilton, ON Canada; 7 Centre for Evaluation of Medicines, St. Joseph’s Health Care, Hamilton, ON Canada; 8 Biostatistics Unit, St. Joseph’s Healthcare-Hamilton, Hamilton, ON Canada; 9 Michael G. De Groote School of Medicine, McMaster University, Hamilton, ON Canada

**Keywords:** Endocrinology, Medical research, Oncology

## Abstract

Acute lymphoblastic leukemia (ALL) is the most common type of childhood cancer. Treatments of ALL predispose survivors to obesity, which increases the risk of cardiovascular disease and diabetes. The hallmark of obesity is excess fat mass, and adiposity is a superior predictor of cardiometabolic risk when compared to Body Mass Index (BMI), yet clinical measures of adiposity in children are lacking. The Tri-Ponderal Mass Index (TMI) (kg/m^3^) is a more accurate adiposity measure compared to BMI z-score in the general pediatric population. This cross-sectional study aimed to validate TMI as an adiposity measure against DEXA scan-derived adiposity, and to compare it to BMI z-score, in pediatric ALL survivors. This study was a retrospective chart review of pediatric ALL survivors diagnosed between 2004 and 2015 at McMaster Children’s Hospital, a tertiary pediatric center in Ontario, Canada. One hundred and thirteen patients (Female n = 55, 48.70%) were included, and adiposity was measured using DEXA scans. Exploratory partial correlations and linear regression analyses were adjusted for age, sex, ethnicity, and ALL risk status. Both TMI and BMI z-score correlated with the DEXA-measured fat mass percentage (FM%) (partial correlation TMI versus FM% r = 0.56; *p* value < 0.0001; BMI z-score versus FM% r = 0.55; *p* value < 0.0001). In regression analyses, the association of TMI was not inferior to BMI z-score in assessing adiposity (TMI versus FM% estimated unstandardized B 0.80, 95% CI 0.56, 1.02; *p* value < 0.0001; BMI z-score versus FM% (unstandardized B 0.37, 95% CI 0.26, 0.49; *p* value < 0.0001). The TMI is a useful clinical adiposity-specific measure in survivors of pediatric ALL.

## Introduction

Acute Lymphoblastic Leukemia (ALL) accounts for 25% of all childhood cancers, making it the most common pediatric malignancy with a worldwide incidence of 1–4.75/100,000^1,2^.

These children undergo 2–3 years of multimodal chemotherapy, including treatment with corticosteroids^3^. Some children with the high-risk disease would also receive craniospinal irradiation or hematopoietic stem cell transplantation^4–6^. Survival of children with ALL has reached around 90% in some high-income countries^7–9^. However, these survivors are at risk of developing cardiometabolic disorders including obesity, metabolic syndrome, type 2 diabetes mellitus, and cardiovascular diseases. Obesity is one of the significant drivers of adverse outcomes and, together with cardiometabolic disorders, can impact the quality of life and life expectancy of these survivors^1,10–17^.

The causes of obesity in this population are multifactorial and include steroids and radiotherapy, obesity at diagnosis, and sedentary lifestyle. Obesity can be associated with metabolic abnormalities including dyslipidemia and dysglycemia^12,13,18,19^. While weight management interventions have been attempted during and after therapy, they have had a limited success^20^.

The expansion of the adipose tissue compartment is the hallmark of obesity; epidemiological evidence suggests that adiposity is a robust predictor of cardiovascular diseases and type 2 diabetes mellitus risk markers in children compared to the most common clinical diagnostic measure of obesity, the Body Mass Index (BMI)^21,22^.

The protocols used for treating children with ALL recommend regular assessments of bone health using Dual-Energy X-ray Absorptiometry (DEXA) as the gold standard^17,23,24^. While DEXA scans measure the bone mass, they also incorporate measurements of the adipose tissue mass. As adiposity is an essential driver of cardiometabolic risk, adipose tissue assessment may help predict future cardiometabolic risk and is an attractive approach to identify at-risk survivors.

However, DEXA scans are expensive, require designated space for the specialized equipment, and need trained personnel to use the machinery and interpret the scans^25–28^. Defining clinical adiposity measures can help measure fat mass changes during and post-treatment. It may also help evaluate the response to future adiposity management interventions in survivors, as these interventions are currently lacking^20^.

The Tri-Ponderal Mass Index (TMI) was recently validated as a more accurate measure of adiposity than Body Mass Index (BMI) and BMI z-score in the general pediatric population and survivors of childhood brain tumors^29,30^. Importantly, the TMI utilizes the height and weight measurements recorded in routine clinical settings and considers childrens’ growth patterns by adjusting weight to height cubed (kg/m^3^) versus the conventional use of height squared in BMI-based calculations (kg/m^2^)^29^.

The aim of this study was to validate TMI as a clinical measure of adiposity against DEXA compared to the BMI z-score in childhood ALL survivors.

## Results

### Population characteristics

The details of participants are reported in Table 1. Of the 113 participants included in this study, 55 (48.70%) were female. Both males and females had similar age at diagnosis (male 6.20 ± 5.10 years; female 5.10 ± 3.50 years) and at the first DEXA scan post-therapy (male 10.40 ± 5.60 years; female 9.90 ± 4.90 years). The majority of participants were Caucasian (n = 97, 85.80%; female n = 47, 85.50%).

**Table 1:** Study population characteristics.

Variables	Total (n = 113) (mean ± SD)	Males (n = 58) (mean ± SD)	Females (n = 55) (mean ± SD)
Age at diagnosis (years)	5.70 ± 4.40	6.20 ± 5.10	5.10 ± 3.50
Age at post-therapy DEXA scan (years)	10.20 ± 5.30	10.40 ± 5.60	9.90 ± 4.90
**Ethnicity**			
Caucasian	97 (85.80%)	50 (86.20%)	47 (85.50%)
Middle Eastern	8 (7.10%)	3 (5.20%)	5 (9.10%)
South Asian	3 (2.70%)	2 (3.40%)	1 (1.80%)
Mixed ethnicity	2 (1.80%)	1 (1.70%)	1 (1.80%)
Hispanic	1 (0.90%)	1 (1.70%)	0
East Asian	1 (0.90%)	1 (1.70%)	0
African	1 (0.90%)	0	1 (1.80%)

*SD* standard deviation.

**Table 2:** Anthropometric and DEXA data in survivors of childhood ALL.

Variables	Total (n = 113) (mean ± SD)	Males (n = 58) (mean ± SD)	Females (n = 55) (mean ± SD)
Height (cm)	136.20 ± 25.40	139.50 ± 28.30	132.80 ± 21.70
Height SDS	0.02 ± 1.10	0.10 ± 1.10	− 0.07 ± 1.10
Weight (kg)	41.10 ± 28.00	46.90 ± 33.60	37.00 ± 19.50
Weight SDS	0.03 ± 1.10	0.17 ± 1.20	− 0.10 ± 0.90
BMI percentile (%)	64.90 ± 33.60	66.50 ± 32.80	63.20 ± 34.70
BMI z-score	0.60 ± 1.30	0.70 ± 1.40	0.60 ± 1.30
TMI (kg/m^3^)	14.80 ± 3.00	14.90 ± 3.00	14.80 ± 3.00
**DEXA data**			
DEXA-Fat Mass (%)	33.40 ± 8.00	32.50 ± 7.50	34.20 ± 8.40
Fat Content (kg)	14.90 ± 12.60	16.70 ± 15.10	13.00 ± 8.90
Lean Body Mass (kg)	26.20 ± 15.80	29.90 ± 18.90	22.20 ± 10.50
Bone Density (g/cm^2^)	0.62 ± 0.19	0.63 ± 0.20	0.60 ± 0.18
Bone Mineral Apparent Density (g/cm^2^)	0.09 ± 0.03	0.10 ± 0.02	0.09 ± 0.04
Bone Mineral Content (g)	1075.40 ± 603.30	1196.50 ± 709.90	947.80 ± 436.50

*TMI* triponderal mass index, *BMI* body mass index, *SD* standard deviation.

The anthropometric data and DEXA scan measures of body composition are reported in Table 2. Males were taller and had a higher weight when compared to females (height: male SDS 0.10 ± 1.10, female SDS -0.07 ± 1.10; weight: male SDS 0.17 ± 1.20, female SDS − 0.10 ± 0.90). While BMI percentiles were similar between males and females (male 66.50 ± 32.80; female 63.20 ± 34.70), the BMI z-scores were higher in male participants when compared to females (male 0.70 ± 1.40; female 0.60 ± 1.30). The TMI measures were similar in both sexes (male 14.90 ± 3.00; female 14.80 ± 3.00).

**Table 3:** Leukemia subtype, risk status, and treatment details.

Variables	Frequency (n, %) n = 113	Males (n, %) n = 58	Females (n, %) n = 55
**Leukemia subtype**			
B cell	102 (90.20)	49 (84.50)	53 (96.40)
T cell	11 (9.80)	9 (15.50)	2 (3.60)
**Risk status**			
Standard Risk	78 (69.00)	34 (58.60)	44 (80.00)
Intermediate Risk	1 (0.90)	1 (1.70)	0
High Risk	32 (28.30)	22 (37.90)	10 (18.20)
Very High Risk	2 (1.80)	1 (1.70)	1 (1.80)
**Treatment protocol**			
DFCI 11–001	25 (22.10)	12 (20.70)	13 (23.60)
DFCI 05–001	77 (68.20)	42 (72.40)	35 (63.60)
DFCI 2000–01	11 (9.70)	4 (6.90)	7 (12.70)

*ALL* Acute Lymphoblastic Leukemia, *DFCI* Dana Farber Cancer Institute Protocol.

Eight females (14.50%) and eight males (13.80%) had a TMI > 85th percentile signifying excess adiposity. The female TMI cut-off was 17.20 kg/m^3^ (range 17.20–26.50) and male TMI cut-off was 17.70 kg/m^3^ (range 17.70–26.40).

### ALL treatments

The details of the ALL subtypes, risk status, and treatment protocols are reported in Table 3. B-cell ALL was the most common subtype (n = 102, 90.20%). Most ALL cases were in the standard risk category (n = 78, 69.00%), while 32 (28.30%) were in the high-risk category. All 113 patients were treated with Dana-Farber Cancer Institute (DFCI) protocols between 2004 and 2015^31–34^. The majority of patients were treated according to DFCI ALL Consortium Protocol 05–001 (DFCI 05–001) approved and activated in 2005 (n = 77, 68.20%)^31–34^. Steroid regimens in the treatment protocols are reported in Supplementary Table S1. Five patients received craniospinal irradiation (male n = 3, 1650.00 ± 653.80 cGy; female n = 2, 1500.00 ± 424.30 cGy).

### Associations of TMI and BMI z-score with DEXA-based adiposity measures

Spearman’s correlation analyses were conducted to assess the correlation between body mass and adiposity measures and unadjusted and age, sex, ethnicity, and ALL risk status-adjusted partial correlations are reported in Table 4. The TMI correlated strongly with BMI z-score (r = 0.81; *p* value < 0.0001) and both TMI and BMI z-score demonstrated a positive correlations with DEXA-derived FM% (TMI r = 0.56, *p* value < 0.0001; BMI z-score r = 0.55, *p* value < 0.0001).

**Table 4:** Partial correlations of TMI and BMI z-scores with DEXA-based Fat Mass Percentage (adjusted for age, sex, ethnicity and risk status).

Variable	BMI z-score	*p* value	FM%	*p* value
**Unadjusted correlations**				
TMI	0.81	< 0.0001	0.54	< 0.0001
BMI z-score	–	–	0.57	< 0.0001
**Partial correlations – adjusted for age, sex, ethnicity, and risk status**				
TMI	0.82	< 0.0001	0.56	< 0.0001
BMI z-score	–	–	0.55	< 0.0001

*TMI* triponderal mass index, *BMI* body mass index, *FM*% fat mass percentage.

To further assess TMI associations with DEXA-based adiposity, multivariable regression analysis with adjustments for age, sex, ethnicity, and ALL risk status was conducted (Table 5). The TMI was associated with the DEXA-based fat mass percentage (FM%; unstandardized B 0.80; 95% CI 0.56, 1.02; *p* value < 0.0001). A similar regression analysis was repeated with the BMI z-scores and demonstrated its association with the FM% (unstandardized B 0.37; 95% CI 0.26, 0.49; *p* value < 0.0001).

**Table 5:** Multivariable linear regression analyses for TMI associations in pediatric ALL survivors adjusted for age, sex, ethnicity, and risk status. Abbreviations: TMI, triponderal mass index; BMI, body mass index; FM%, fat mass percentage.

Variables	Unstandardized B coefficient	95% CI		*p* value	Model summary
		Lower bound	Upper bound		Adjusted R^2^
**Dependent variable: BMI z-score**					
TMI	1.80	1.65	2.05	< 0.0001	0.67
**Dependent variable: FM%**					
TMI	0.80	0.56	1.02	< 0.0001	0.32
BMI z-score	0.37	0.26	0.49	< 0.0001	0.32

**Figure 1:**
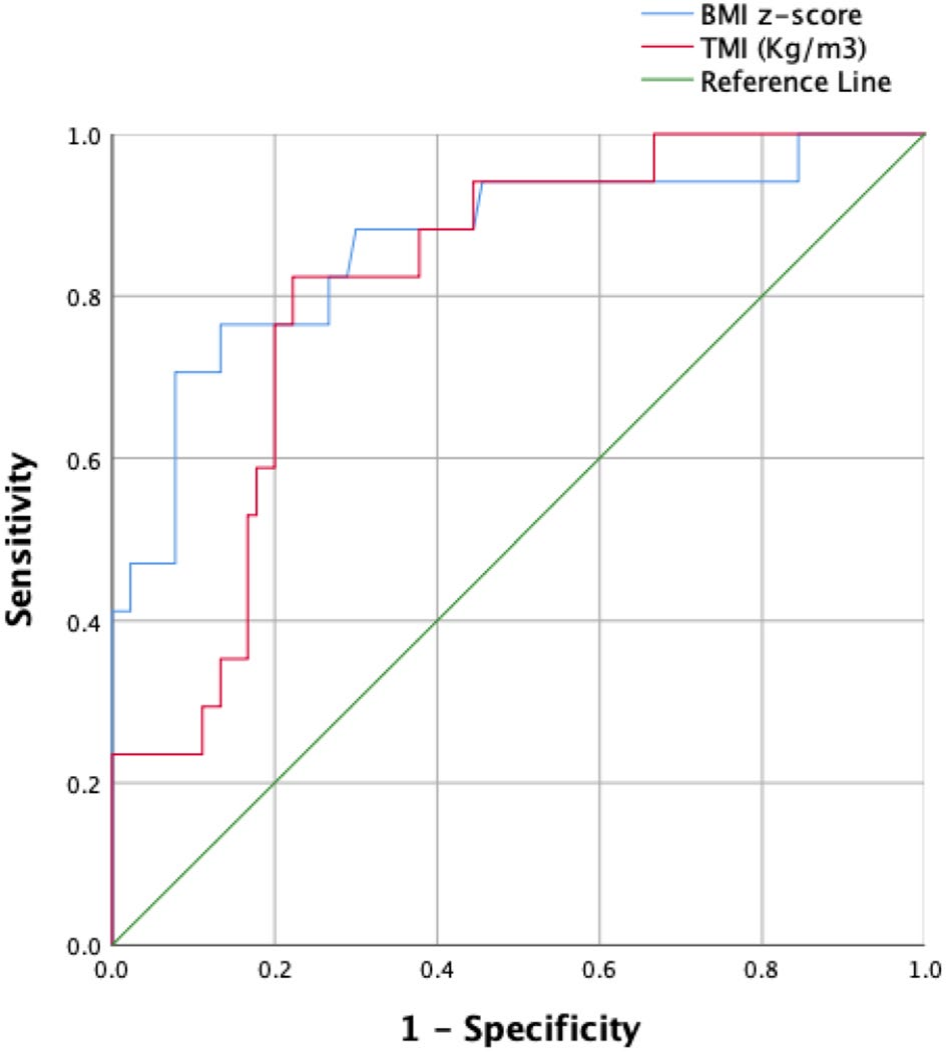
The ROC curve for the TMI and BMI z-score association with the DEXA-based fat mass percentage (FM%).

To further compare the associations of the TMI and BMI z-scores with DEXA-based adiposity, we generated the Receiver Operating Characteristic (ROC) curve (Fig. 1) and calculated the Area Under the Curve (AUC). The analysis demonstrated that both TMI and BMI z-score were excellent tests to predict the DEXA-based adiposity (TMI: AUC 0.81, 95% CI 0.71–0.91, *p* value < 0.0001; BMI z-score: AUC 0.86, 95% CI 0.76–0.97, *p* value < 0.0001).

Furthermore, the TMI was associated with the BMI z-score (unstandardized B 1.80, 95% CI 1.65, 2.05; *p* value < 0.0001).

Taken together, TMI and BMI z-score is associated with DEXA-based total adiposity measures in pediatric survivors of ALL.

## Discussion

The advent of novel ALL therapies has transformed the life expectancy of children with ALL over the past few decades^35^. With the reduction in premature mortality, there has been a widened focus on mitigating the burden of morbidities of ALL and its treatment^20^.

Some of the most significant morbidities reported in survivors include adverse cardiometabolic health outcomes such as type 2 diabetes and cardiovascular diseases^17,36^. As adiposity is a predictor of adverse cardiovascular and metabolic outcomes, reliable biological and clinical markers of adiposity are urgently needed to identify and target survivors with interventions that mitigate cardiometabolic risk. This study demonstrated that TMI, similar to BMI z-score, is significantly associated with DEXA-based total adiposity measures in pediatric ALL survivors. However, as an adiposity-specific measure, TMI has several important advantages when compared to BMI-based measures to estimate adipose tissue mass.

TMI has a stronger association with total adiposity in the general pediatric population compared to BMI z-score, the latter being inaccurate in estimating adiposity and misclassifying physically advanced children as being overweight or obese^29,37–40^.

Also, TMI is associated with central adiposity measures, especially waist-to-height ratio, in the general pediatric population and survivors of childhood brain tumors^30,41,42^. While our data demonstrate that TMI and BMI z-scores are comparable in their association with total adiposity, TMI is a more specific measure of total adiposity. This specificity may offer an added advantage in the childhood ALL survivors when estimating the adipose mass. The adipose tissue-specific nature of TMI, along with it being sex-specific and age- and puberty-independent offers a useful clinical tool that can be tracked longitudinally to assess changes in adiposity in survivors. Importantly, it can also be used to track responses to interventions to manage adiposity in this population.

An important question is whether TMI can predict cardiometabolic outcomes across the lifespan. While TMI is inferior or equal to BMI z-scores in detecting insulin resistance, it is a better predictor of metabolic syndrome in children than BMI^43–45^, and is positively associated with systolic and diastolic blood pressure in obese youth, although the latter association has not been consistently reported^46–49^.

Also, childhood BMI and TMI can predict adult obesity, type 2 diabetes, increased carotid intima-media thickness, and elevations in Low-Density Lipoprotein in the general population^43^. However, the use of TMI as a tool to predict long-term cardiometabolic outcomes in ALL survivors needs further characterization, as there are likely differences between ALL patients and the general population regarding the mechanisms driving adiposity in these groups.

Pediatric ALL patients are exposed to corticosteroids throughout their treatment^32–34^, which leads to excess adiposity, increased appetite, weight gain, and decreased bone mineral density^17,50–52^. Importantly, steroid treatment leads to the expansion of the total fat mass and is not associated with the redistribution of fat from the limbs to the trunk^53^, This makes TMI a valid tool to assess adiposity in survivors, as most of the steroid-driven weight gain is related to adipose tissue expansion rather than generalized growth, including muscle and bone compartments pattern seen with exogenous obesity^53^.

One of the strengths of this study is that we report on the association of TMI with total adiposity in ALL survivors, a population that has not been previously studied. We have also validated the TMI against DEXA, the gold standard in adiposity measurement.

One of the study's limitations is that no central adiposity measures were included in the analysis, and this should be the goal of future studies. The correlation of TMI with waist-to-height ratio and a lesser extent waist-to-hip ratio, as central adiposity measures have been documented^30^, and would be quite essential to replicate in the ALL population that is already at higher risk of adverse cardiometabolic disorders when compared to the general population. The study's cross-sectional nature is another limitation, as the evolution of TMI measures over time would be essential to assess. The inclusion of pubertal staging and its correlation with TMI in ALL survivors is important, as adiposity increases throughout puberty, particularly in females^39^.

## Conclusion

In conclusion, this study reports the association of TMI and BMI z-score with DEXA measures of adiposity in survivors of ALL. This study's findings provide further evidence that TMI is an accurate and feasible clinical marker of adiposity in this population that is at high risk of cardiometabolic disorders. Also, the TMI is advantageous as an age-independent and sex-specific measure of adiposity than BMI z-scores. This allows the use of constant cut-offs to determine adiposity in different pediatric populations, including childhood ALL survivors. There is a need to validate TMI use in ALL survivors to decide whether it can predict long-term cardiometabolic outcomes.

## Methods

### Participants

This study was a cross-sectional study of pediatric ALL survivors who were diagnosed between August 2004-June 2015 at McMaster Children’s Hospital, a tertiary pediatric academic center in Hamilton, Ontario, Canada. One hundred and thirteen patients (n = 55 female, 48.70%) had DEXA data available for analysis. The study was a retrospective chart review approved by the Hamilton Integrated Research Ethics Board that exempted the study from consent requirements, as the retrospective chart review design involved the deidentification of the data after collection. The study procedures were performed following the relevant guidelines and legal regulations.

Data were obtained from patient charts through electronic medical record systems. The anonymized data were collected at the end of treatment for each patient, including age, age at diagnosis, sex, ethnicity, ALL subtype, ALL risk status, and treatment data. Anthropometric measures, including height and weight, were collected, with the height measured to the closest 0.1 cm and weight measured to the nearest 0.1 kg. For the DEXA scan data bone mineral content, bone density, bone mineral apparent density, total fat (kg), fat mass percentage (FM%), and lean body mass were retrieved from the patient’s first available DEXA scan post completion of therapy. The mean duration between the completion of treatment and the DEXA scan was 22.30 ± 29.70 months.

### BMI and TMI calculations

The BMI and TMI were calculated using height and weight measurements. BMI was calculated as the weight (kg) divided by the height squared in meters (m^2^)^29^, while TMI was calculated as the weight (kg) divided by height cubed in meters (m^3^)^29^. BMI percentiles were calculated using the World Health Organization Growth Charts for Canada^54^ and BMI z-scores were calculated using the Calculator from the Centres for Disease Control and Prevention anthropometric Z-scores calculator^55^. Fat Mass Percentage (FM%) were obtained from DEXA scans (Hologic densitometer (Discovery A; Hologic, Inc, Bedford, Massachusetts). Puberty data were unavailable.

### Statistical analyses

Statistical analyses were performed using SPSS Version 25.0 for Macintosh^56^. Data are presented as frequencies (%) for categorical variables and means (SD) for continuous variables. Box plots and visual inspections were used to identify outliers for removal from the analysis. Data distribution was assessed for normality using the Shapiro–Wilk and Kolmogorov–Smirnov tests^57.^ The age, BMI z-score, TMI, and FM% variables were log-transformed to account for non-normal distributions.

Unadjusted as well as partial correlations of the associations between TMI, BMI, and DEXA-based adiposity measures were performed that were adjusted for age, sex, ethnicity, and ALL risk status. Multivariable linear regression analyses were conducted to assess the associations of total body mass and adiposity with TMI. The dependent variable was the FM% or BMI z-score, and the independent variables were TMI, age, sex, ethnicity, and ALL risk status. Results were reported as standardized and unstandardized beta coefficients with 95% confidence intervals and associated *p* values with statistical significance set at α of 0.05. The ROC curve and AUC calculations were used to further examine the association of TMI with DEXA-based adiposity^58^.

## Supplementary Information

Supplementary Information.

## Data Availability

The data for the current study used for statistical analysis are available from the corresponding author upon reasonable justification.
