# Supplementary material for: The triponderal mass index as a measure of adiposity in pediatric survivors of acute lymphoblastic leukemia: a cross-sectional study
Source: Sci Rep. 2022 Jan 26;12:1404. doi: 10.1038/s41598-022-05236-5 (PMC8792003; doi:10.1038/s41598-022-05236-5)
Supplement: Supplementary file 1 — Supplementary Information. [file 41598_2022_5236_MOESM1_ESM.docx]

**Supplementary Table S1:** ALL therapy schema for dosage and duration of steroid use and cranial radiation

| **Therapy Phase** | **Risk Status** | **Steroid/ Radiation** | **Average Cumulative Dosage** |
| --- | --- | --- | --- |
| DFCI 200-01 | | | |
| Induction (4 weeks) | All patients | Prednisone  Or methylprednisolone | 40mg/m^2^  1.3mg/kg if <0.6m^2^  30mg/m^2^/day  1.3mg/kg if <0.6m^2^ |
| CNS and Intensification Therapy (33 weeks) | SR patients | Intrathecal Therapy Hydrocortisone | Age 1-1.99: 594mg  Age 2-2.99: 792mg  Age >3: 990mg |
|  |  | Prednisone  Or Dexamethasone | 2200mg/m^2^  71.5 mg/kg if <0.6m^2^  330mg/m^2^  11mg/kg if <0.6m^2^ |
|  | HR patients | Cranial Radiation  CNS+ patients | 1200cGy  1800cGy |
|  |  | Prednisone  Or Dexamethasone | 6600mg/m^2^  220mg/kg if <0.6m^2^  990mg/m^2^  27.5 mg/kg if < 0.6m^2^ |
| Continuation (70 weeks) | All patients | Intrathecal Therapy Hydrocortisone | Age 1-1.99: 162mg  Age 2-2.99: 216mg  Age >3: 270mg |
|  |  | Prednisone  Or Dexamethasone | 2200mg/m^2^  71.5mg/kg if <0.6m^2^  330 mg/m^2^  11mg/kg if <0.6m^2^ |
| DFCI 2005-01 | | | |
| Prophase (3 days) | All patients | Methylprednisolone | 96mg/m^2^ |
| Induction (4 weeks) | All patients | Prednisone or Prednisolone  Or Methylprednisolone | 1160mg/m^2^  928mg/m^2^ |
|  |  | Intrathecal Therapy Hydrocortisone | Age 1-1.99: 9mg  Age 2-2.99: 12mg  Age >3: 15mg |
| Consolidation I (3 weeks, 7 weeks for HR patients) | SR & HR patients | No steroids | |
|  | VHR patients | Dexamethasone | 90mg/m^2^ |
| CNS Therapy (3 weeks) | SR patients | Dexamethasone | 30mg/m^2^ |
|  |  | Intrathecal Therapy Hydrocortisone | Age 1-1.99: 54mg  Age 2-2.99: 72mg  Age >3: 90mg |
|  | HR & VHR patients | Dexamethasone | 90mg/m^2^ |
|  |  | Intrathecal Therapy Hydrocortisone | Age 1-1.99: 54mg  Age 2-2.99: 72mg  Age >3: 90mg |
|  |  | Cranial Radiation | 1200cGy |
| Consolidation II (27 weeks) | SR patients | Dexamethasone | 270mg/m^2^ |
|  |  | Intrathecal Therapy Hydrocortisone | Age 1-1.99: 27mg  Age 2-2.99: 36mg  Age >3: 45mg |
|  | HR & VHR patients | Dexamethasone | 810mg/m^2^ |
|  |  | Intrathecal Therapy Hydrocortisone | Age 1-1.99: 27mg  Age 2-2.99: 36mg  Age >3: 45mg |
| Continuation (~70 weeks until 104 weeks (24 months) of CCR) | All patients | Dexamethasone | 720mg/m^2^ |
|  |  | Intrathecal Therapy Hydrocortisone | Age 1-1.99: 35mg  Age 2-2.99: 47mg  Age >3: 58mg |
| DFCI 11-001 | | | |
| Prophase (3 days) | All patients | Methylprednisolone | 96mg/m^2^ |
| Induction (4 weeks) | All patients | Prednisone or prednisolone  Or Methylprednisolone | 1160mg/m^2^  928 mg/m^2^ |
|  |  | Intrathecal Therapy Hydrocortisone | Age 1-1.99: 9mg  Age 2-2.99: 12mg  Age >3: 15mg |
| Consolidation I (3 weeks, 7 weeks for VHR patients) | SR & HR patients | No steroids | |
|  | VHR patients | Dexamethasone | 90mg/m^2^ |
| CNS Therapy (3 weeks) | SR patients | Dexamethasone | 30mg/m^2^ |
|  |  | Intrathecal Therapy Hydrocortisone | Age 1-1.99: 54mg  Age 2-2.99: 72mg  Age >3: 90mg |
|  | HR & VHR patients | Dexamethasone | 90mg/m^2^ |
|  |  | Intrathecal Therapy Hydrocortisone | Age 1-1.99: 54mg  Age 2-2.99: 72mg  Age >3: 90mg |
|  |  | Cranial Radiation | 1200cGy |
| Consolidation II (27 weeks) | SR patients | Dexamethasone | 270mg/m^2^ |
|  |  | Intrathecal Therapy Hydrocortisone | Age 1-1.99: 27mg  Age 2-2.99: 36mg  Age >3: 45mg |
|  | HR patients | Dexamethasone | 810mg/m^2^ |
|  |  | Intrathecal Therapy Hydrocortisone | Age 1-1.99: 27mg  Age 2-2.99: 36mg  Age >3: 45mg |
| Continuation (~70 weeks until 104 weeks (24 months) of CCR) | All patients | Dexamethasone | 720mg/m^2^ |
|  |  | Intrathecal Therapy Hydrocortisone | Age 1-1.99: 35mg  Age 2-2.99: 47mg  Age >3: 58mg |

Abbreviations: DFCI, Dana Farber Cancer Institute; CNS, Central Nervous System; SR, Standard Risk; HR, High Risk; VHR, Very High Risk; CCR, Continuous Complete Remission.
